# Carotid blood flow is correlated with cardiac output but not with arterial blood pressure in porcine fecal peritonitis

**DOI:** 10.1186/cc9490

**Published:** 2011-03-11

**Authors:** T Correa, A Reintam Blaser, J Takala, S Djafarzadeh, M Vuda, M Dünser, S Mathias Jakob

**Affiliations:** 1 University Hospital Bern - Inselspital and University of Bern, Switzerland

## Introduction

Cerebral blood flow may be impaired in sepsis [1]. The objective of this study is to evaluate whether and how carotid blood flow (CBF) depends on cardiac output and mean arterial blood pressure in abdominal sepsis.

## Methods

Thirty-two anesthetized pigs (weight: 40.3 ± 3.7 kg (mean ± SD)) were randomly assigned (*n *= 8 per group) to a nonseptic control group (CG) or one of three groups in which resuscitation was initiated 6, 12 or 24 hours after induction of fecal peritonitis (instillation of 2 g/kg autologous feces). In the treatment groups, resuscitation was performed for 48 hours according to the Surviving Sepsis Campaign. The CG was observed for 72 hours. CBF (carotid artery; ultrasound Doppler flow), cardiac output (intermittent thermodilution) and arterial blood pressure (MAP) were measured at 6-hour intervals. Pearson correlation were performed between CBF index (CBFI) and cardiac index (CI) and MAP, respectively, both in individual animals and in pooled septic and control groups.

## Results

**Figure 1:**
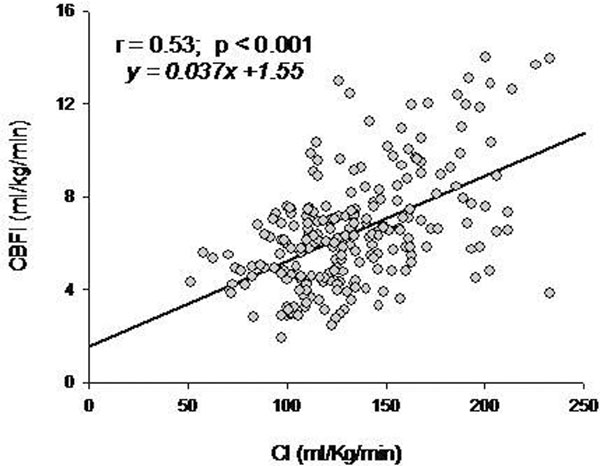
**Correlation between CBFI and CI**.

**Figure 2:**
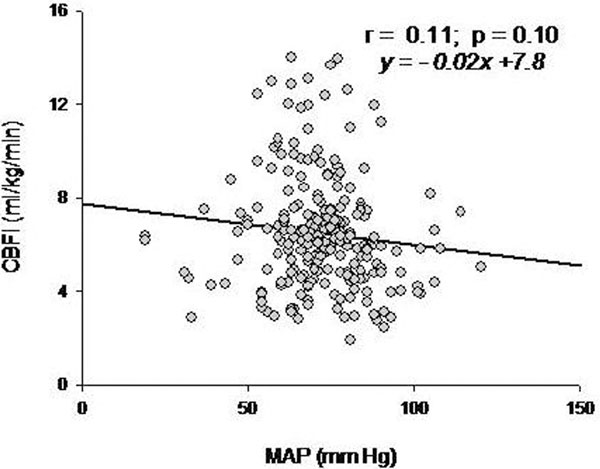
**Correlation between CBFI and MAP**.

Altogether 227 measurements were obtained during sepsis and 128 in controls. In septic animals, CBFI and CI (*r *= 0.53, *P *< 0.001; Figure 1) but not CBFI and MAP correlated (Figure 2). In controls, CBFI and MAP correlated weakly and inversely (*r = *-0.246, *P *= 0.005; data not shown).

## Conclusions

Under the experimental conditions, increasing systemic blood flow but not blood pressure has the potential to improve CBF.
